# The Social Norm of Hematopoietic Stem Cells and Dysregulation in Leukemia

**DOI:** 10.3390/ijms23095063

**Published:** 2022-05-03

**Authors:** Geoffrey Brown

**Affiliations:** School of Biomedical Sciences, Institute of Clinical Sciences, College of Medical and Dental Sciences, University of Birmingham, Edgbaston, Birmingham B15 2TT, UK; g.brown@bham.ac.uk; Tel.: +44-(0)121-414-4082

**Keywords:** hematopoietic stem cells, leukemia, leukemia stem cells, lineage fate, oncogenes

## Abstract

The hematopoietic cell system is a complex ecosystem that meets the steady-state and emergency needs of the production of the mature blood cell types. Steady-state hematopoiesis replaces worn out cells, and the hematopoietic system is highly adaptive to needs during, for example, an infection or bleeding. Hematopoiesis is highly integrated and the cell hierarchy behaves in a highly social manner. The social tailoring of hematopoietic stem cells to needs includes the generation of cells that are biased towards a cell lineage; these cells remain versatile and can still adopt a different pathway having made a lineage “choice”, and some cytokines instruct the lineage fate of hematopoietic stem and progenitor cells. Leukemia stem cells, which may well often arise from the transformation of a hematopoietic stem cell, sustain the hierarchy of cells for leukemia. Unlike hematopoietic stem cells, the offspring of leukemia stem cells belongs to just one cell lineage. The human leukemias are classified by virtue of their differentiating or partially differentiating cells belonging to just one cell lineage. Some oncogenes set the fate of leukemia stem cells to a single lineage. Therefore, lineage restriction may be largely an attribute whereby leukemia stem cells escape from the normal cellular society. Additional antisocial behaviors are that leukemia cells destroy and alter bone marrow stromal niches, and they can create their own niches.

## 1. Introduction

From studies of the cellular origin of acute myeloblastic leukemia (AML), leukemia stem cells (LSCs) sustain disease [1,2]. Like hematopoietic stem cells (HSCs), LSCs are a rare and self-renewing subpopulation of cells and they generate a hierarchy of differentiating or partially differentiating leukemia cells. They are also mostly responsible for leukemia relapse. A longstanding view about the nature of cancer is that the cells outperform their normal counterparts because the gene mutations that are the cause of cancer accelerate the rate of cell division, and/or inhibit the normal controls on cell proliferation. The transcription factor MYC is overexpressed in 60–70% of the human solid cancers and hematopoietic malignancies. Overexpression correlates with cell proliferation and acts to release the brakes on cell cycle, via the inactivation of cell cycle inhibitors (reviewed in [3]). Similarly, the *HRAS*, *KRAS*, and *NRAS* genes are frequently mutated in human solid cancers and hematopoietic malignancies. Aberrant RAS function, via constitutive activation, is associated with hyper-proliferation [4]. Some cancer cells outperform their normal counterpart by failing to undergo apoptosis. For example, the BCL-2 protein, which is anti-apoptotic, is highly upregulated in many cancers [5].

However, are increased proliferation and/or survivability primary attributes to LSCs whereby these cells might gain an advantage? The cancer stem cell theory states that most, if not all, cancers are sustained by cancer stem cells (CSCs) [1,2]. For many solid tumors, CSCs are still somewhat elusive regarding their nature and frequency within a cancer [6]. Estimates of frequency vary from rare up to 25%. Therefore, we do not know whether CSCs divide much more frequently than their normal counterpart, by being less quiescent. Nor do we know whether CSCs are better survivors than normal stem cells because the avoidance of apoptosis by cancer cells has been largely determined for cell populations as a whole. Additionally, cancer cells accumulate multiple mutations that include gene amplifications, chromosomal aberrations, aneuploidy, and microsatellite instability. This has been attributed to a loss of genome stability and the acquisition of a mutator phenotype [7]. Insults to the genome that dysregulate proliferation and/or survivability might merely add to a more fundamental oncogene-mediated dysregulation of the behavior of CSCs.

A tissue-specific stem cell produces a variety of cell types and this is an important consideration to how oncogenic transformation might influence the behavior of a stem cell. This review focuses on hematopoiesis to examine how the needs of an organism are met by the integrated and social production of the different cell types. Regarding HSCs, they, and their offspring, are versatile as to how they “choose” to follow developmental pathways. This review then examines whether LSCs are similarly versatile, and argues that LSCs and their offspring are restricted to just one cell lineage.

## 2. Hematopoiesis Meets Demands

A prompt increase in the production of a particular type of mature cell is needed in response to an emergency, such as infection, inflammation, and injury, because each cell type has a specialized function. It is well established that the conduct of hematopoiesis is highly adaptive to the needs of an organism during invasion by a pathogen to ensure an organism’s survival. For a particular pathogen and as an immune response develops in steps, the production of a type of cell is needed. For example, neutrophils provide the front line of defense against a bacterial infection and they are consumed in large quantities. Hematopoiesis adapts very rapidly to ensure the immediate availability of more neutrophils, whereby emergency granulopoiesis meets this demand [8]. The de novo production of neutrophils is increased substantially from the enhanced proliferation of bone marrow myeloid precursor cells. Hematopoietic cytokines ensure neutrophil production because they are important to the survival and proliferation of the cells that are developing towards neutrophils. They include interleukin (IL)-3, IL-6, granulocyte colony-stimulating factor (G-CSF), and granulocyte/macrophage colony-stimulating factor (GM-CSF). From studies of chemotherapy-induced neutropenia, a rise in G-CSF is a key regulator of increased neutrophil production and G-CSF enhances their function. It also mobilizes HSCs from bone marrow into the blood and influences dendritic cell activation and T-cell function [9]. During emergency granulopoiesis, HSCs and hematopoietic progenitor cells (HPCs) respond appropriately by veering towards myeloid differentiation. HSCs and HPCs cells express the Toll-like pattern recognition receptors (TLRs) for pathogens. For human CD34+ bone marrow cells, which are a mixture of HSCs and HPCs, TLR ligation promoted myeloid differentiation at the expense of lymphoid differentiation (reviewed in [8]). A plausible suggestion is that the engagement of TLRs also stimulates the proliferation of HSCs and HPCs.

There needs to be a balanced production of cell types by HSCs during steady-state hematopoiesis. Too many of one type of cell or a lack of another would not serve tissue homeostasis. Steady-state hematopoiesis differs from hematopoiesis under stress conditions because there are substantial increases in the levels of cytokines locally and systemically during an emergency. Therefore, it is likely that there are differences between the control of steady-state versus emergency hematopoiesis. In particular, HSCs, HPCs, their maturing offspring, and the bone marrow cells that support hematopoiesis are collectively a complex ecosystem whereby the need is to self-regulate the appropriate replenishment of each blood cell type. In this regard, an important principle is that cells behave in a social manner, to the overall benefit of a tissue, by influencing one another. This general principle was established in 1954 from studies of fibroblasts that had migrated out from chick embryo heart explants onto a glass substratum. When they collided their locomotion decreased, their movement became random, and the cell density in the space between the explants became stable. The fibroblasts had self-regulated to become a contact inhibited monolayer [10]. More recent studies have shown that the traffic signals to social cell migration in a multicellular setting are an interplay of the properties of the individual cells and intercellular interactions that involve pseudopod formation, via secreted chemicals, and collapse during collisions [11]. In addition to the above reactive response of hematopoiesis to an invasive pathogen, there is a social context to steady-state hematopoiesis, including the behavior of HSCs, as follows.

### 2.1. Tailoring of the Setup of HSC Compartment

Hematopoiesis in adult mice has been mostly studied in the context of an emergency scenario and less is known about the setup of steady-state. The transfer of bone marrow cells and purified HSCs into a lethally irradiated mouse, and examination of their capacity to reconstitute the cell lineages, has been used to examine the generation of hematopoietic cells and homeostasis. There are concerns about the use of this approach because the reconstituting cells are seeded into a hematopoietic cell vacuum, as depleted by irradiation, and there is also possible damage to the niches that support hematopoiesis. Even so, a study of mouse long-term HSCs (LT-HSCs) has shown that the HSC compartment is flexible to need because LT-HSCs can be functionally reprogrammed [12]. LT-HSCs cells were isolated at 2 and 3 weeks after transplantation into a lethally irradiated mouse and re-transplanted into secondary recipients. Their myeloid versus lymphoid engraftment capacity was compared to that of freshly isolated LT-HSCs by examining HPC subsets. Mouse bone marrow multipotent HPCs that lack lineage markers and that express the Sca-1 antigen and the c-kit receptor for stem cell factor (termed LSK) were divided into sub-sets of multipotent progenitors (MPP) that included myeloid-biased MPP2 (Flk2−, CD150+, CD48+) and MPP3 (Flk2−, CD150−, CD48−) and lymphoid-primed MPP4 (Flk2+, CD150−, CD48+/−). MPP2 and MPP3 have extensive and persistent granulocyte/macrophage potential, whereas that of MPP4 is transient. MPP3 show a dominant myeloid granulocyte/macrophage output in colony-forming methylcellulose assays, whereas that of MPP2 is extensively megakaryocyte. As compared to the control LT-HSCs, regenerating LT-HSCs had lost their self-renewal activity and overproduced myeloid-biased MPPs to rebuild hematopoiesis in the first instance. Regenerating LT-HSCs then recovered their capacity for normal engraftment as seen from the restoration of the production of lymphoid-primed MPP4. In essence, myeloid-biased MPPs were transiently overproduced from LT-HSCs during hematopoietic cell regeneration.

### 2.2. Subsets of Lineage-Biased/Affiliated HSCs

From marking HSCs and lineage tracking studies, using either Sleeping Beauty transposons or *Tie2-Cre* endogenous labeling, HSCs make a limited contribution to native hematopoiesis [13,14]. From the above studies of regenerating LT-HSCs, the investigators had argued that myeloid-biased MPP2s and MPP3s and lymphoid-primed MPP4s combine to support and control native blood cell production [12]. Moreover, a new approach to classifying mouse HSCs is based on the existence of subsets that have particular lineage biases [15]. From transplantation studies, subsets also include HSCs biased towards megakaryocytes, identified by their cell surface expression of the megakaryocyte-restricted CD41 (alphaIIb integrin, platelet GPIIb) [16] or the platelet marker von Willebrand factor. The latter cells required thrombopoietin for their maintenance [17]. Mouse HSCs are lineage-affiliated by virtue of subsets that express a receptor for a lineage-affiliated cytokine at their cell surface. Subsets of LT-HSCs and short-term HSCs (ST-HSCs) express the receptor for macrophage colony-stimulating factor ((M-CSF), 20%) and the fms-like tyrosine kinase 3 ((Flt3), 7%), which binds a myeloid/lymphoid affiliated cytokine. In total, 13% of LT-HSCs and 19% of ST-HSCs express mRNA for the receptor for erythropoietin (Epo) [18]. The lineage potentials of human adult bone marrow HSCs (CD34+, CD38−) and HPCs (CD34+, CD38+) were mapped for single cells using a cell assay and a wide range of cytokines. The cells that predominated were either multipotent cells or unipotent cells with a myeloid or lymphoid potential [19].

Importantly, M-CSF and Epo instruct myeloid and erythroid fate, respectively. When injected intravenously into mice, recombinant M-CSF increased the activation of the myeloid-associated transcription factor (TF) PU.1 in LT-HSCs and the proportion of HSCs with a myeloid bias. These findings were confirmed by in vitro culture experiments [20]. Epo guides multipotent HPCs towards an erythroid fate, thereby decreasing myeloid output [21]. HSCs are master responders to Epo during chronic erythroid stress, as seen from studies using Epo transgenic mice. In this scenario, enhanced cell division of HSCs was observed and these HSCs were tailored to the demand for erythrocytes. They exhibited a committed erythroid progenitor profile, as revealed by an increase in erythroid related TFs, for example, GATA-1, and a 5-fold increase in the expression of *Klf1*, which has a GATA-1 preferred site [22]. The lineage fate of cells that are developmentally downstream of HSCs is also instructed by cytokines. M-CSF commits progenitors with granulocyte/macrophage potentials to a macrophage fate, whereas GM-CSF and G-CSF drive these progenitors towards granulocytes [23,24].

The HSC compartment is a heterogeneous population of cells. That HSCs directly acquire unique lineage affiliations and that the compartment is organized in this way, is proactive towards HSCs displaying a social behavior. It has been proposed that lineage biased subsets play a crucial role during both native and emergency hematopoiesis [12], and an increase in the level of M-CSF and Epo presumably increases the mature cell output from the appropriately biased HSC.

### 2.3. HSC Behavior and Feedback Control from the Level Mature Cells

Organisms have an enormous steady-state capacity to make erythrocytes, with adult humans making more than 2.5 million erythrocytes per second and adult mice making around 7000 erythrocytes per second [25]. The extent to which the production of erythrocytes can be adjusted to produce a large wave to meet an urgent demand has been studied extensively by tail bleeding mice to render them anemic. In this case, the HSC compartment is responsive to the level of mature erythrocytes as tail-vein bleeding led to the proliferation and self-renewal of HSCs in both the bone marrow and spleen. Therefore, the regulators of HSC status are able to sense and respond to the native level of erythrocytes [26].

Additionally, hematopoiesis is reprogrammed during a response to anemia (reviewed in [27]). Proliferation of HSCs in the spleen is important to replace erythrocytes in anemic mice because it has been known for over 50 years that the bone marrow erythroid output is added to by increasing splenic erythropoiesis. Anemic mice exhibit splenomegaly with increased iron uptake. A new view on the spleen-mediated increase to erythrocyte production is that bone marrow ST-HSCs migrate to the spleen and the actions of Hedgehog ligands and bone morphogenic protein BMP4a specify a ‘stress erythroid fate’. The stress burst-forming unit-erythroid cells (BFU-E) produced in the spleen are responsive to BMP4a, whereas bone marrow BFU-E are unresponsive. Stress erythropoiesis also plays a key role during an inflammatory response to maintain erythropoiesis because inflammation inhibits the steady-state production of erythrocytes to accommodate the need to increase myelopoiesis. In this case, inflammation induces stress erythropoiesis [27]. Epo plays a central role in driving stress erythropoiesis [28] and, as above, can instruct erythroid fate within multipotent HPCs.

## 3. HSC Affiliation to a Cell Lineage

**Figure 1:**
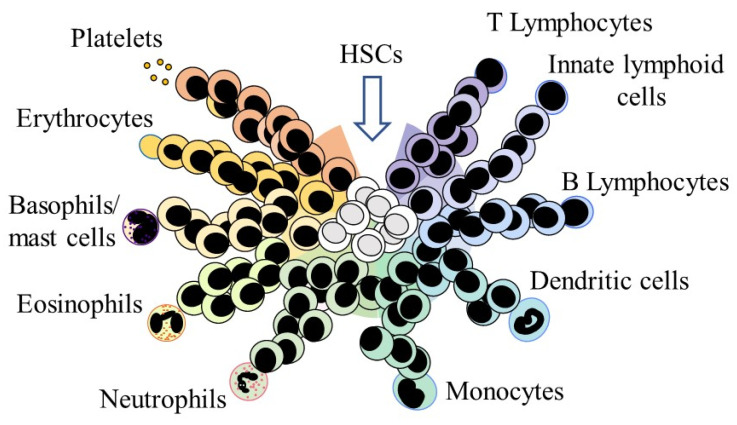
A continuum model for hematopoiesis. Hematopoietic stem cells (HSCs) are a mixture of cells with different lineage signatures, as shown by the different colors for the lineage-affiliated HSCs that are within the colored arc for the HSC compartment. Some HSCs have ‘chosen’ a lineage directly from a spectrum of the end cell options. Even so, they and hematopoietic progenitor cells remain versatile. There are particular close relationships between the cell lineages and HSCs and hematopoietic progenitor cells that have “chosen” a pathway can step left or right into a different pathway to give rise to a different cell type.

Affiliation to a single cell lineage can occur early within HSCs, which is very different to the view depicted in the conventional tree-like models of hematopoiesis. In these longstanding models, the lineage options of developing HSCs are progressively restricted stepwise, via a series of binary decisions and intermediate HPCs, towards a single-lineage committed cell. By contrast, a continuum model for hematopoiesis depicts that a spectrum of all lineage options is available to HSCs, and they can directly acquire an intrinsic bias towards/affiliation to a developmental pathway (Figure 1) [29]. Whether there is a social order to how the options arise in the first instance within HSCs is uncertain. It has been argued that HSCs with a megakaryocyte bias reside at the apex of hematopoiesis [17]. The continuum model does not prescribe a preferred route to each mature cell type because the progression of HSC development is continuous, broad, and versatile, as follows.

### HSCs and Their Offspring Remain Versatile

HSCs that have ‘chosen’ a developmental pathway are still able to adopt a different pathway. This aspect of the social behavior of HSCs is supported by a number of findings. For cells that are developing along the erythroid, neutrophil/macrophage, and lymphoid pathways, the trajectories are broad, as revealed by the RNA expression maps obtained from the sequencing of more than 1600 single mouse HSCs and HPCs. Developing cells may then move to the left or right of a chosen pathway [30]. In this case, a progressive differentiation process that is flexible precedes the establishment of a final and stable fate. In the continuum model, the near neighbor developmental options were inferred from the shared use of TFs and other characteristics (Figure 1). For example, the TF GATA-1 is shared by the megakaryocyte and erythroid pathways, and human HSCs that are megakaryocyte-primed can step sideways towards erythropoiesis [31]. Second, progression towards a major pathway and a mature end cell type is a continuous process for HSCs and their offspring. This was revealed by constructing developmental trajectories, from single-cell RNA sequencing and differentiation outcomes, for human HSCs/their immediate progeny (Lin−, CD34+, CD38−) versus the more differentiated HPCs (Lin−, CD34+, CD38+). Human HSCs/their immediate progeny were observed to be a continuously connected entity, and the findings separated the more differentiated HPCs into distinct HPCs for each of the major hematopoietic cell types [32]. A striking example of lineage versatility is shown by progenitor cells that have progressed some way towards becoming T-cells. They give rise to macrophages when cultured in the presence of M-CSF, and can also give rise to dendritic cells and natural killer cells [33,34].

## 4. The Genesis of Leukemia

To return to fibroblasts, at least two genomic insults are required for the conversion of primary rat embryo fibroblasts to cells that seed tumors that grow to a large size. The insults include, for example, the activation of a *RAS*-like and a *MYC*-like gene [35]. Similarly, at least two genomic insults are needed for the genesis of childhood acute lymphoblastic leukemia (ALL), as shown from twin concordance studies [36]. The first genomic insult gives rise to a preleukemic cell that is, however, compatible with a normal downstream hematopoietic development in the majority of the cases, as illustrated by the presence of this clone in up to 5% of healthy newborns [37]. The second insult, likely triggered by an environmental stimulus, will transform this preleukemic clone into a full-blown leukemia. By virtue of their capacity to self-renew, HSCs are at risk of transformation, which is in keeping with the stem cell theory of cancer [1,2]. Therefore, does one of the two oncogenic insults to an HSC lead to the generation of LSCs that behave in an antisocial manner, and, if so, in what way?

### 4.1. Some Leukemias Have a Specific Mutational Signature

As mentioned above, MYC and BCL2 protein overexpression and RAS activation are oncogenic events that occur in many different cancers. By contrast, certain oncogenes are very different because they are prevalent in a particular malignancy. The reciprocal t (9;22) chromosomal translocation in human chronic myeloid leukemia (CML) fuses the *BCR* gene to the *ABL* proto-oncogene [38,39], resulting in the Philadelphia chromosome (Ph+) and BCR-ABLp210 oncoprotein. Patients who develop Ph+ precursor B-cell ALL have the fusion gene *BCR-ABLp190* and BCR-ABLp190 oncoprotein, and BCR-ABLp190 transcripts are found in the leukocytes of normal individuals and the cells are presumed to be pre-leukemic [40,41,42]. Eighty percent of cases of B-cell precursor ALL in infants (<1 year of age) have a chromosomal rearrangement resulting in the *MLL-AF4* fusion gene [43]. Twenty-five percent of cases of childhood B-ALL have the fusion gene *ETV6-RUNX1* (known also as *TEL/AML1*) [44]. Activation of the *LMO2* gene, by chromosomal translocations, is exclusive to T-cell leukemias [45]. In the case of MALT lymphomas, the main chromosomal translocations are t (11;18) (q21;21) and t (14;18) (q32;q21), and involve *MLT/MALT1* [46]. Signature mutations are not restricted to hematological malignancies because they extend to, for example, sarcomas. A t (11;22) (q24;q12) chromosomal translocation fuses the *EWS* gene onto chromosome 22 with the *FLI1* gene onto chromosome 11 in over 90% of cases of Ewing’s sarcomas [47]. Synovial sarcoma is characterized by the translocation t (X;18) (p11;q11), which fuses the *SS18 (SYT*) gene on chromosome 18 with *SSX1*, *SSX2*, or rarely *SSX4* on chromosome X [48].

### 4.2. The Cell of Origin for Some Leukemias

Forty years ago, it was clear that CML arises from the transformation of an HSC and that the clone causes a very large increase in the number of granulocytes [49]. B-cell precursor ALL in infants is an aggressive disease with pro-B cells accumulating in the bone marrow. However, there is evidence to support the view that a primitive cell that lies upstream of a fetal liver B cell progenitor is the cell of disease origin. A suggestion is that a fetal liver lymphoid-primed multipotent progenitor (LMPP) is the cell of origin of infant B-cell precursor ALL [50]. LMPPs are lympho-myeloid stem cells; they can produce neutrophils, monocytes, B cells, and T cells and fail to produce significant erythroid cells and megakaryocytes [51]. Childhood B-ALL has been thought to arise in a B-cell committed progenitor [52], and the leukemia cells express the early stage B-cell antigen CD19. By contrast, there is evidence to support the view that the cell of origin is a more primitive HSC-like cell [53,54,55] whereby the offspring of the transformed cell are restricted to the B-cell lineage. The origin of childhood B-ALL is still unclear [56]. A longstanding view is that the subtypes of chronic lymphocytic (CLL) are a monoclonal expansion arising from the transformation of antigen-experienced B-cells. However, self-renewing HSCs are aberrant in CLL, and this cell has been proposed as the origin of CLL [57,58].

From the above, a striking observation is that certain genes are often mutated in a particular leukemia. For the leukemias, there is evidence to support the view that the cell of origin is an HSC, or a primitive HSC-like cell. However, the progeny of LSCs resemble a non-pathological differentiated or partially differentiated cell that belongs to just one cell lineage. One way of reconciling these findings is that the specific genetic insult specifies the lineage fate of the LSCs.

## 5. Some Oncogenes Restrict LSCs and Their Offspring to a Single Cell Lineage

**Figure 2:**
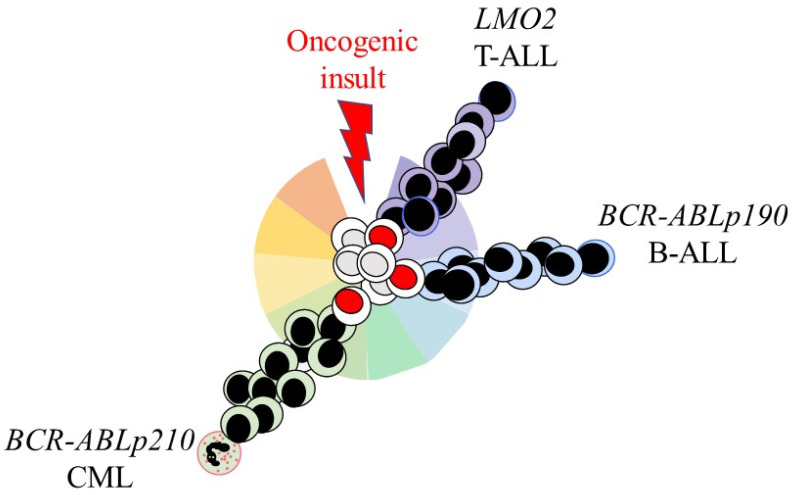
Some oncogenes set the lineage fate of LSCs and their offspring. Expression of the oncogenes *BCR-ABLp210*, *BCR-ABLp190*, and *LMO2* was restricted to hematopoietic stem and progenitor cells in transgenic mice by means of the Sca1 promoter. This led to the development of myeloid, B-cell, and T-cell leukemia.

As considered above, the *BCR-ABLp210*, *BCR-ABLp190*, and *LMO2* oncogenes drive human CML, Ph+ B-cell ALL, and T-cell ALL. The influence of these oncogenes on HSCs/HPCs was investigated by restricting their expression to stem cell antigen 1 (Sca1) + HSCs/HPCs in transgenic mice. The oncogenes were active solely within the leukemia-initiating cells/LSCs and, therefore, not essential for the survival/proliferation of the more mature lineage-affiliated leukemia cells. The action of the above oncogenes replicated the human disease, and, in particular, restricted the offspring of LSCs to a cell lineage (Figure 2).

Sca1-directed expression of human *BCR-ABLp210* within HSCs/HPCs in the transgenic mice (*Sca1-BCR-ABLp210)* resulted in a myeloid leukemia (Figure 2) [59,60]. Further studies of the transgenic mice showed that the action of *BCR-ABLp210* is via epigenetic reprograming of HSCs/HPCs towards granulocytes. For the *Sca1-BCR-ABLp210* mice, the DNA methyltransferase 1 was upregulated within HSCs/HPCs, and a granulocyte malignancy developed in *Sca1-Dnmt1* transgenic mice [60]. When *BCR-ABLp190* was targeted to HSCs/HPCs in transgenic mice, via *Sca1-BCR-ABLp190*, the mice developed a precursor B-cell ALL that resembled the human disease and at a low penetrance (13%). Transgenic mice that were double *Sca1-BCR-ABLp190* and *Pax5* (a B cell TF) +/− were generated. Ninety percent of these mice developed precursor B-cell ALL with a much shorter latency, and the remaining wild-type *Pax5* allele accumulated genomic alterations. The investigators concluded that *Sca1-BCR-ABLp190* had set the cell lineage of HSCs/HPCs, and that *BCR-ABLp190* and loss of *Pax5* are required for precursor B-cell ALL [61]. Targeting the expression of *LMO2* to different hematopoietic cell compartments in transgenic mice, via the Sca1 promoter, revealed that LMO2 can set the identity of various cell types to give rise to an aggressive and highly disseminated T-cell ALL. The cell populations that were targeted included HSCs/HPCs, the pro-B cell stage of development, and germinal centre B-cells [62]. These findings argue against the leukemia cells belonging to just one lineage because the oncogene had targeted a differentiated type of cell.

As mentioned above, the fusion gene *ETV6-RUNX1* is associated with childhood B-ALL and two oncogenic insults are needed for leukemia. It has been proposed that the second insult arises from an abnormal response to infection [36]. In transgenic mice, *ETV6-RUNX1* expression was targeted to pro-B cells and the mice were exposed to natural infections. However, the mice did not develop leukemia. The *KDM5C* gene, which encodes an H3K4me3 and H3K4me2 demethylase, is missense mutated in mouse *ETV6-RUNX1* B-ALL and human relapse *ETV6-RUNX1* B-ALL. Hence, loss of function of the H3K4 demethylase was investigated [63]. Transgenic mice still failed to develop B-cell ALL when *KDM5C* loss-of-function was introduced into the B cell compartment of mice that expressed *ETV6-RUNX1*. By contrast, when *ETV6-RUNX1* expression was initiated in HSCs/HPCs in transgenic mice and the mice were exposed to natural infections they developed T-cell (35%) and B-cell (6%) ALL. The introduction of both ETV6-RUNX1 expression in HSCs/HPCs and *KDM5C* loss-of-function led to 22% of the transgenic mice developing B-ALL when kept in a special pathogen-free environment. From the above findings, the investigators concluded that *ETV6-RUNX1* can trigger T-cell and B-cell leukemias and that the lineage identity of the leukemia cells is set by the second ‘hit’, with both ‘hits’ having to occur in the HSC/early HPC compartment [64].

## 6. The Antisocial Interplay between LSCs and Bone Marrow Niches

HSCs and HPCs reside in specialized niches in the bone marrow. The niches that HSCs and HPCs reside in are heterogeneous and the interplay with various stromal cells is important to the social norm of steady-state hematopoiesis [65]. This aspect of the social behavior of HSCs has been written about extensively. The cytokine support to HSC survival, proliferation, and development is also made available within niches, as presented by niche cells. Leukemia cells are as dependent as their normal counterparts on cytokines for their survival and proliferation. AML cells have not become independent of cytokine support because interleukin (IL)-3 and GM-CSF are needed to support the growth of AML precursors [66]. Similarly, the survival of leukemic B-cell precursors requires IL-3, IL-7, and stromal cell support [67].

Much like hematopoiesis, leukemia is an interplay between LSCs and their offspring and the surrounding bone marrow microenvironment [68]. These interactions are viewed as important to treatment options, but they are as yet not well understood. Changes to the niches for steady-state hematopoiesis have also been implicated in the initiation of leukemia, and, for example, a deficiency of retinoic acid receptor γ in the microenvironment leading to myeloproliferative syndrome [69]. From mouse models involving the deletion and overexpression of various molecules, changes to the microenvironment have been observed to either initiate and/or promote leukemogenesis, but it is still unclear whether the engineered changes cause human leukemia [70]. A popular view is that leukemia and all cancers progress in a Darwinian-like manner [71], and changes to the bone marrow microenvironment might play a role in the selection and propagation of clones, though whether this is the case is as yet unclear [72].

### 6.1. LSCs Disruption to Bone Marrow Niches

Cytopenia occurs often in patients with leukemia because normal hematopoiesis is disrupted. Xenografting of mouse femurs with human CD34+ AML cells and normal HSCs/HPCs (CD34+ cells) has shown that these cells share bone marrow niches. Dose escalation studies revealed that the higher doses of normal cells outcompeted LSCs, and displacement of LSCs from niches allowed their replacement with normal HSCs/HPCs. Therefore, LSCs do not have a superior affinity for the niches [73]. Even so, the bone marrow is viewed as containing a limited number of saturable niches and competition between LSCs and normal HSCs will have an impact on normal hematopoiesis

Leukemia cells change the bone marrow microenvironment by destroying and altering niches and by changing the balance of niches. Osteoblastic lineage cells are able to sustain HSCs. For a Notch-driven mouse model of T-cell ALL that recapitulates the human disease, leukemia cells were isolated from a primary host and injected into secondary recipients. They infiltrated across bone marrow with accumulated disease leading to a complete loss of mature osteoblastic cells and less efficient HSC function [74]. In a transgenic model of CML, the chronic phase CML cells stimulated mesenchymal stem and progenitor cells to overproduce osteoblastic lineage cells, but they were functionally altered and accumulated as pro-inflammatory myelofibrotic cells. This remodeling compromised support to HSCs, whereas LSCs were resilient to the change to the osteoblastic lineage cells [75]. In a mouse model of *MLL-AF9* AML, the development of AML induced sympathetic neuropathy, which led to an expansion of perivascular mesenchymal stem and progenitor cells primed for osteoblastic differentiation at the expense of the periarteriolar cells, which are important to the maintenance of HSCs [76].

**Figure 3:**
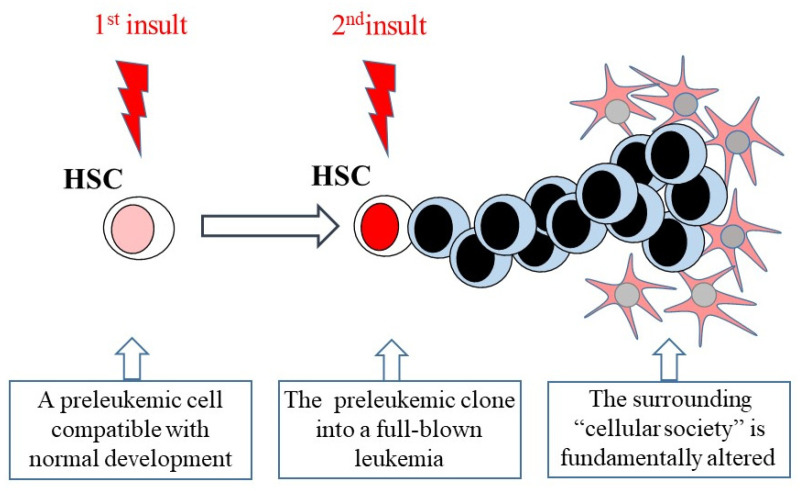
The genesis of the pathology of leukemia. The preleukemic cell that arises from the 1st insult is compatible with normal development. It is transformed into a leukemic clone by the second insult. This clone gives rise to a full-blown leukemia. The LSCs and/or their progeny fundamentally alter their surrounding “cellular society”.

Whereby LSCs produce cells that are restricted to developing along a pathway, there is presumably an increased demand for particular bone marrow niches to ensure the offspring’s survival. A further aspect to the antisocial behavior of leukemia cells is that they can create abnormal bone marrow vascular niches that disrupt the bone marrow niches for normal hematopoiesis [77]. This was revealed by using a severe combined immunodeficiency mouse xenograft model of human Nalm-6 pre-B acute ALL cells and human HPCs (CD34+ve cells from cord and peripheral blood). These cells were engrafted intravenously, and tracked by dynamic in vivo imaging. In the leukemic mice and over time, the engrafted normal HPCs declined. The engrafted Nalm-6 leukemia cells had located to, and proliferated within, bone marrow niche sites that were positive for the cytokine stromal cell-derived factor-1 (SDF-1). These sites overlapped with the peripheral vascular niches for HPCs. The leukemia cells created abnormal bone marrow niches, as they disrupted SDF-1 expression at the niche sites, and secreted the HPC growth factor stem cell factor (SCF) (Figure 3). SCF is a chemo-attractant for HPCs [78]. In the xenograft model, engrafted HPCs had sensed a gradient of SCF, migrated towards the malignant vascular niches, and were sequestered. When HPCs were previously established in normal bone marrow niches, by introducing normal HPCs 12 to 16 weeks earlier than Nalm-6 cells, the malignant niches also competed for population by HPCs. A migration similar to that seen for Nalm-6 leukemia cells and upregulation of SCF signal was seen in the bone marrow when bone marrow aspirates from ALL and AML patients were engrafted in nonobese diabetic-severe combined immunodeficiency mice. Leukemia cells can, therefore, usurp normal niche control on HPCs to cause dysfunction.

### 6.2. LSC Invasion of Distant Tissues

As cancer cells become more malignant they are able to break out from the boundaries of their tissue environment, enter and exit the blood stream or the lymphatic system, and invade distant tissues. Metastasis means ‘new place’, and CSCs are largely responsible for solid tumor metastatic disease. Leukemia cells infiltrate the spleen, but this might not be considered as metastatic behavior in terms of the leukemia cells retaining the capacity of their normal counterparts to migrate and invade. On the other hand, it has been argued that, from the anatomy of the leukemias, as for solid tumors, the dissemination of leukemia cells is a metastatic process [79]. However, perhaps the key question is do LSCs behave differently from HSCs in terms of their capacity to migrate and invade? Though mostly resident in a specialized niche in the bone marrow, HSCs can be found in peripheral blood, and ST-HSCs are mobile as seen from the stress erythropoiesis that occurs in the spleen. LSCs might, therefore, be not too different from HSCs regarding an ability to migrate, but this matter has still to be resolved.

## 7. Concluding Remarks

HSCs behave in a highly social manner to meet the demands of an organism for a cell type, as needed. They reside in specialized niches that control their survival, self-renewal, and whether they differentiate. The existence of sub-populations of HSCs that are lineage-affiliated is tailored to meeting the demand for a particular cell type. Whether HSCs acquire a bias towards/affiliation to a developmental pathway by a process that is stochastic or deterministic has been debated for some time [80]. Recent mathematical modeling of how HSCs veer towards a lineage envisages a high order of multi-stability within HSCs [81], and HSCs gradually acquire uni-lineage priming [32,82,83] where noise and bursting gene expression play key roles. This and multi-stability fit with a continuum model to show how HSCs adopt a pathway, and are contradictory to a bi-stable, tree-like and dichotomous model. Albeit, and as above, M-CSF and Epo can instruct/guide a lineage affiliation within HSCs and multipotent HPCs, respectively, which is important to the social norm of hematopoiesis. HSCs that have ‘chosen’ a fate remain versatile, and presumably this ensures that hematopoiesis is highly socially responsive to a particular demand.

LSCs do not conform to the social norms of HSCs. Some leukemias clearly arise in HSCs, for example, CML. Some leukemias have been thought to arise in later lineage-committed progenitor cells, but there is evidence to support an origin in a cell that is developmentally earlier. If we presume that many of the leukemias arise in an HSC, the offspring of LSCs, unlike those of normal HSCs, belong to one cell lineage. Indeed, we classify the different leukemias by affiliating the leukemia cells to a cell lineage. Some oncogenes set the lineage fate of LSCs and their progeny. For example, the oncogene *BCR-ABLp210* is the mutational signature to CML, this myeloid disease arises from an HSC, and a myeloid disease arose when the expression of BCR-ABLp210 was limited to HSCs/HPCs in transgenic mice. The differentiating leukemic cells did not require maintenance of expression of the oncogene for their survival and proliferation, and, as brought to attention in a commentary, the oncogene’s action is “hands-on” within LSCs and “hands-off” regarding the differentiating offspring [84]. What might go wrong to change the social norm of LSCs? Perhaps the oncogene fixes a lineage burst of gene expression or cooperates to lead to the adoption of a pathway. Leukemia cells are also antisocial because they disrupt the bone marrow niches that provide support to HSCs. For the future, it is important to resolve how all of this comes together to add to our understanding of the cardinal features of LSCs and, in turn, develop treatments that target LSCs.
